# Isolated Intracranial Rosai-Dorfman Disease

**DOI:** 10.1155/2016/1972594

**Published:** 2016-02-02

**Authors:** Md. Taufiq, Abul Khair, Ferdousy Begum, Shabnam Akhter, Md. Shamim Farooq, Mohammed Kamal

**Affiliations:** ^1^Department of Pathology, Square Hospitals Ltd., Dhaka 1205, Bangladesh; ^2^Department of Neurosurgery, Green Life Medical College and Hospital, Dhaka 1205, Bangladesh; ^3^Department of Pathology, Bangabandhu Sheikh Mujib Medical University (BSMMU), Dhaka 1000, Bangladesh; ^4^Department of Pathology, Dhaka National Medical College, Dhaka 1100, Bangladesh

## Abstract

*Background*. Rosai-Dorfman disease (RDD) is a benign histiocytic proliferative disorder of unknown etiology. This rare condition commonly causes massive cervical lymphadenopathy. Intracranial RDD without any nodal involvement is extremely rare.* Case Report*. A young Bangladeshi male complained of bilateral complete blindness with left sided deafness for about three years. There was no lymphadenopathy. MRI and CT scan of brain suggested an inflammatory/neoplastic (?meningioma) lesion located at left parasellar region which extended frontally to encircle both optic nerves and also to left prepontine area. Histopathologically the lesion was diagnosed as RDD. The patient was treated with steroid and significant clinical improvement observed.* Conclusion*. The prognosis of intracranial RDD is not poor. It can be treated with surgery with or without corticosteroids, chemotherapy, and so forth. But as the condition is extremely rare and often misdiagnosed, the clinician, radiologist, and histopathologist should have a suspicion in their mind about the possibility of RDD.

## 1. Introduction

Rosai-Dorfman disease (RDD), also known as sinus histiocytosis with massive lymphadenopathy, is a benign histiocytic proliferative disorder of unknown etiology [1]. It is usually characterized by cervical lymphadenopathy along with systemic features [2]. Extranodal RDD may occur in 43% of cases [3, 4]. Common sites are orbit, head and neck region, upper respiratory tract, skin, bone, testis, and so forth [5, 6]. However, in less than 5% of cases, RDD has been reported in central nervous system [1, 6]. In most of the occasions, the intracranial RDD is associated with nodal involvement [7]. Isolated intracranial RDD without nodal involvement is extremely rare [1, 5]. Approximately 70 of such cases have been published so far [8]. Here we present a case of isolated intracranial RDD with very rare clinical features.

## 2. Case Report

**Figure 1:**
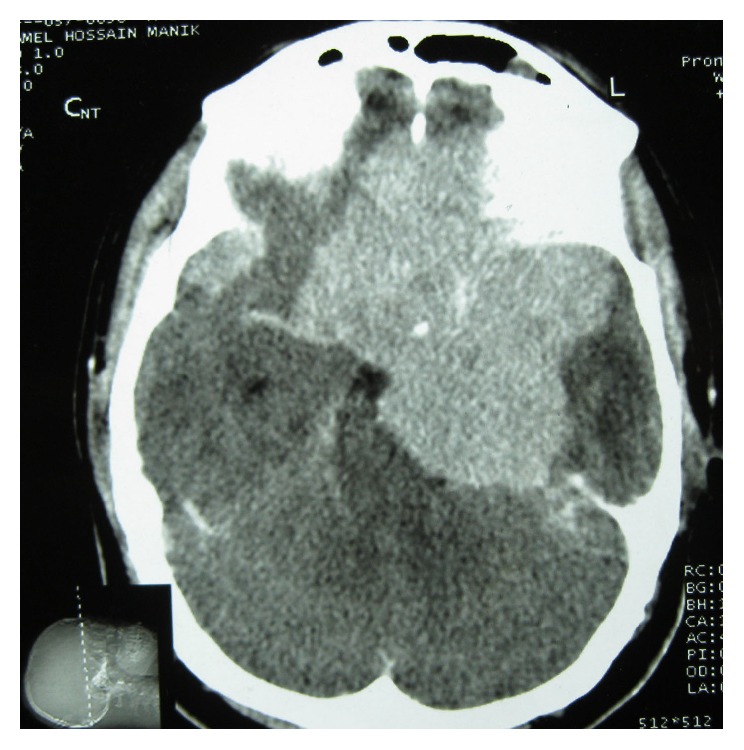
CT scan of brain showing that the lesion has encircled both optic nerves.

**Figure 2:**
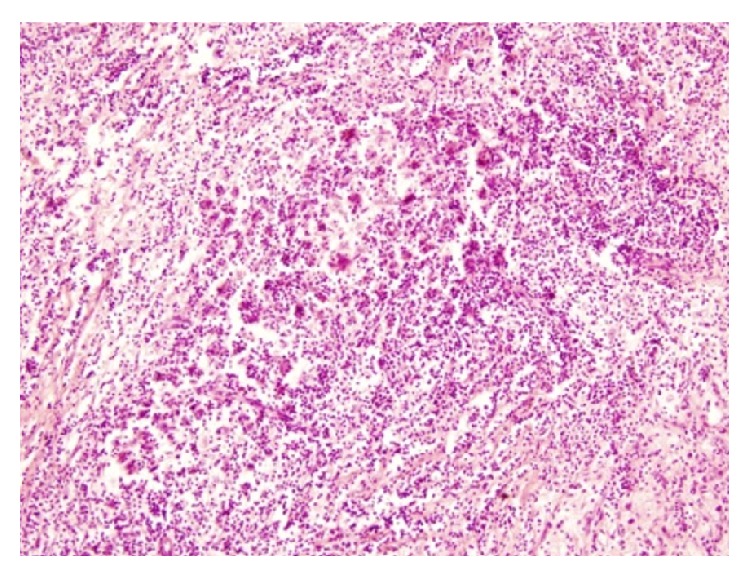
Photomicrograph showing inflammatory nature of the lesion (H&E, ×200).

**Figure 3:**
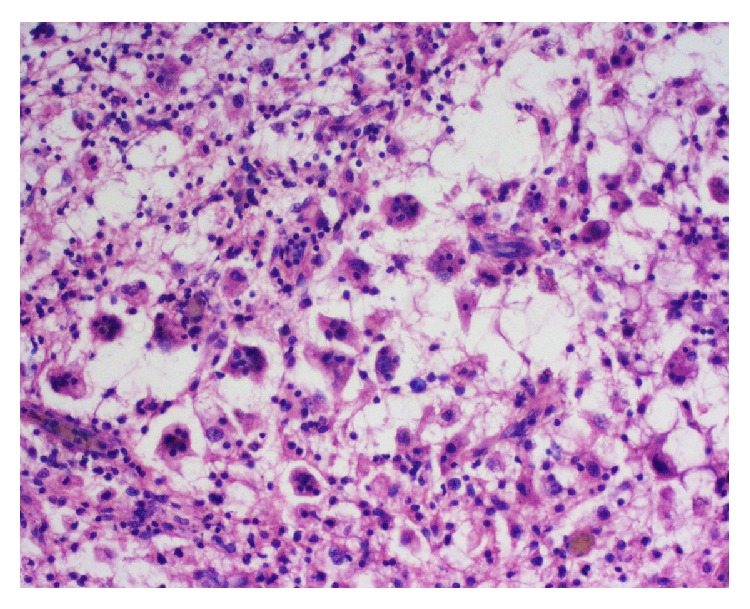
Photomicrograph of the lesion showing many histiocytes, some of which have engulfed intact lymphocytes (H&E, ×400).

**Figure 4:**
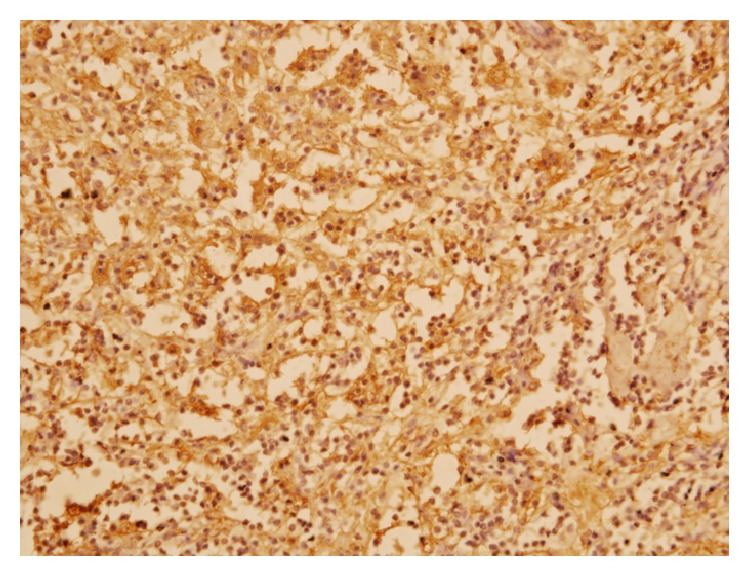
Photomicrograph of immunohistochemistry of the lesion showing positive reactivity of the histiocytes to S-100 protein (S-100, ×400).

A 24-year-old Bangladeshi male, hailing from rural area of Comilla, complained of bilateral dimness of vision (more on left side), headache, nausea, and deafness of left side since early 2008. He consulted ophthalmologists, but there was no improvement. On the contrary, patient gradually became completely blind. Then he was referred to Bangabandhu Sheikh Mujib Medical University (BSMMU), Dhaka. He was normotensive and nondiabetic and had no lymphadenopathy. His blood group was “O” positive. Haematological findings were as follows: haemoglobin 12.8 gm/dL, erythrocyte sedimentation rate (ESR) 14 mm after 1st hour, and total leukocyte count 8.8 × 10^9^/liter. X-ray of chest and paranasal sinuses (occipitomental view) was normal. Audiometric examination revealed hearing sensitivity of right ear within normal limit but no response to any frequency in left ear. Only vibrotactile response was present in left ear. MRI of brain revealed hypointensity lesions alongside of the cavernous sinus and retroocular region of right orbit. Findings were suggestive of inflammatory lesion/neoplastic masses (?meningioma). An endoscopic endonasal transethmoidal approach was made to take biopsy from the lesion. Histopathology of biopsied material revealed portion of optic nerve, fragmented pieces of soft tissue, chronic inflammatory cells, and a single focus of oval cells forming whorled pattern. The lesion was suggested as an inflammatory pseudotumour with a differential diagnosis of lymphoplasmacyte-rich meningioma. Patient was released from hospital with only symptomatic treatment and was advised to come back for a follow-up after three months. Patient failed to attend in time. In the meantime, he received some homeopath treatment but there was no improvement. After one year, he developed frequent fainting, nausea, and vomiting. At the end of 2011, patient came back to Dhaka and was admitted to a private hospital in neurosurgery department. A computed tomography (CT) scan of brain revealed large, irregular, hypodense mass lesions with mild perilesional oedema in left parasellar region on the medial aspect of left temporal lobe, extending in both frontal regions and in both retrobulbar areas encircling both optic nerves. The lesion has also extended in left prepontine area causing mild compression upon the adjacent pons. The final radiological impression was left parasellar meningioma (Figure 1). At this stage, bifrontal craniotomy was done. A whitish, tough, fibrous, relatively avascular, calcified space occupying lesion (SOL) was found. Subtotal removal of the SOL was done and sent for histopathology. Microscopically, the tumour showed hyalinized fibrocollagenous tissue containing nodular collections of foamy histiocytes, lymphocytes, and plasma cells (Figure 2). A few foci showed emperipolesis (lymphophagocytosis by histiocytes) (Figure 3). One of the sections also showed a small focus of meningothelial cells. Immunohistochemically, the emperipolesis histiocytes were positive for S-100 protein (Figure 4) and negative for AE1/AE3 (pan-cytokeratin), epithelial membrane antigen (EMA), vimentin, and estrogen receptor. Finally the tumour was diagnosed as a case of intracranial Rosai-Dorfman disease. The patient was treated with steroid. Initially after surgery, the patient suffered from weakness in his right side with slurring of speech in addition to his previous complete blindness and left sided deafness. However, gradually he was improving. Currently (January 2014) he is able to walk independently and there is a slight improvement of his deafness. His vision, however, did not improve.

## 3. Discussion

Rosai-Dorfman disease (RDD) was first described by Destombes in 1965 and was recognized as a distinct clinicopathological entity by Rosai and Dorfman in 1969 [1]. It usually occurs in children and young adults (mean age of onset 20.6 years) and shows a male sex predilection (male to female ratio 1.4 : 1) [1, 5]. The disease is usually characterized by massive painless bilateral cervical lymphadenopathy along with fever, leukocytosis, high ESR, and polyclonal hypergammaglobulinaemia [2]. Microscopically, lymph nodes show dilated sinuses containing foamy histiocytes and plasma cells. Many of these histiocytes contain intact hematopoietic cells, mostly lymphocytes within their cytoplasm, a phenomenon known as emperipolesis [4–6]. Although not specific, this is a constant feature of RDD [2]. On immunohistochemical examination, these histiocytes are positive for S-100 protein and CD 68 and negative for CD1a [2, 5, 9].

There are some hypotheses regarding the etiology of this rare condition. The histiocytes in RDD may derive from activated macrophages. This activation may be caused by an unusual response of the hematolymphoid system to an immune disorder. Some authors also have suggested a viral influence behind this disease. With in situ hybridization, Epstein-Barr virus and human herpes virus type six were identified in some cases [5].

Extranodal RDD occurs in about 43% of cases [3, 4, 8]. Common sites are orbit, head neck, upper respiratory tract, skin, bone, testis, and so forth [5, 6]. However, less than 5% of cases of RDD may occur in central nervous system with or without nodal involvement. Among these, 75% of cases occur in brain and 25% in spinal cord [1]. Isolated intracranial RDD without nodal involvement is extremely rare. To our knowledge, approximately seventy of such cases have been published so far [8].

Unlike nodal disease, intracranial RDD usually affects adult males in their fourth and fifth decade (mean age 39.5 years which is little higher than nodal RDD) [6, 9]. Usually, it presents as a dura based solitary mass [6]. Multiple lesions are unusual but have been reported [1, 6]. Common locations are suprasellar region, cerebral convexity, parasagittal region, cavernous sinus, and petroclival region [1, 5]. Clinical features depend on location of the tumour and may include headache, epilepsy, and cranial nerve deficit [5, 6].

Radiologically intracranial RDD is commonly mistaken for meningioma [6, 7]. This mistake was also made in the present case. This is because both of the conditions are usually dura based. In T2 weighted MRI, meningiomas show low to high signal intensity [5]. On the other hand, RDD shows hypointensity or isointensity on T1 and T2 weighted images and shows marked homogenous enhancement after contrast administration [7]. On angiograms, meningiomas are commonly hypervascular. In contrast, vascularity of RDD is variable [5]. Other radiological differential diagnoses of intracranial RDD include Langerhans cell histiocytosis, lymphoma, metastatic carcinoma, melanoma, and granulomatous inflammation like tuberculosis and sarcoidosis [5, 6].

The definitive diagnosis of intracranial RDD depends on histopathology and immunohistochemistry. But unfortunately the characteristic histological finding (e.g., emperipolesis) is often obscured by extensive fibrosis that occurs in intracranial RDD. Andriko et al. [3] described eleven cases of isolated intracranial RDD. Out of these, emperipolesis was evident only in seven cases with haematoxylin and eosin stain. In the present case, extensive hyalinized fibrous tissue was present. Only a few foci showed small number of histiocytes with phagocytosed intact lymphocytes (emperipolesis).

This case was initially diagnosed histologically as lymphoplasmacyte-rich meningioma. This is an important differential diagnosis of intracranial RDD. This type of meningioma produces a chronic inflammatory infiltrate which can be confused with infiltrates present in RDD. Immunostain with epithelial membrane antigen (EMA) can differentiate the two entities precisely [3]. The current case is negative for EMA.

Langerhans cell histiocytosis, on the other hand, is another mimicker of intracranial RDD. Eosinophils are also present in this condition. Langerhans histiocytes, when xanthomatous, may resemble histiocytes in RDD. Moreover, immunologically both RDD and Langerhans histiocytes are positive for S-100. However, Langerhans cells have folded nucleus with longitudinal grooves which is absent in RDD histiocytes. Unlike RDD, Langerhans histiocytes are also positive for CD1a [3]. In the current case, the histiocytes are positive for S-100. CD1a reactivity is not done, because the marker is not available. But, morphologically, the histiocytes with engulfed lymphocytes (emperipolesis) are quite different from Langerhans cells.

Ophthalmic manifestations of RDD may occur due to orbital and eyelid involvement, lacrimal gland involvement, optic nerve compressive neuropathy, and uveitis. Among these, orbital manifestation is the commonest [10]. On the other hand, only a few case reports are available in the literature regarding visual impairment due to compression of optic nerve by RDD [11–15].

In the present case, the mass (RDD) extended to the retrobulbar region and has encircled both optic nerves causing compression and complete blindness. Other reported cases have shown that optic nerve compression due to RDD may be unilateral [13, 14] or bilateral [15]. Visual impairment is usually partial [15]. But as seen in the present case, complete blindness may also result. However, all of these cases were not exclusively intracranial like the present case. Extracranial [13] and nodal involvements [12] were present along with optic nerve compression in some of these cases.

Cause of left sided deafness in this patient is not fully understood. Pure tone audiometry reveals sensorineural hearing loss (SNHL) of left ear. The patient showed no response to any frequency of sound wave in that ear. But the hearing sensitivity of right ear is normal.

Sensorineural hearing loss may be caused by lesions in the cochlea or the auditory nerve. It may be congenital, hereditary, or delayed onset. Delayed onset SNHL is usually caused by infection, trauma, or ototoxic medication. But it may also be caused by primary tumours in temporal bone (vestibular schwannoma, squamous cell carcinoma, and paraganglioma) or metastatic tumours to temporal bone (breast, lungs, and prostate) [16].

In this patient, the intracranial mass (RDD) has extended in left prepontine area and has caused mild compression upon the adjacent pons. This may cause damage to auditory nerve pathway and ultimately result in deafness of left ear.

Reported cases of RDD causing hearing loss are extremely rare. Nalini et al. [17] in 2012 described an unusual case of progressive bilateral visual impairment and hearing loss due to extensive intracranial RDD. Yetiser et al. [18], on the other hand, reported two male siblings who presented with sensorineural hearing loss due to intracranial RDD. Ahsan et al. [19] observed another case involving bilateral external auditory canals and middle ear causing hearing loss. This patient also had extensive tracheobronchial tree involvement.

Rosai-Dorfman disease has been treated with surgery, corticosteroids, chemotherapy, and radiotherapy [1, 5, 6]. Among these, surgical excision appears to be most effective especially if there is nerve compression [6, 7]. In the present case, complete resection was not possible due to extensive involvement of adjacent structures by the tumour. The patient is now getting corticosteroids. He is improving gradually. Nowadays, he can walk and can hear better with his left ear.

The prognosis of RDD is not poor even if the disease is intracranial. A review of follow-up data of 43 cases of RDD with CNS involvement showed that 58% of patients were alive [5]. Nine out of eleven patients described by Andriko et al. lived 2–42 months (mean 15 months). No patient had recurrence of the disease even with subtotal resection [3].

## 4. Conclusion

Intracranial Rosai-Dorfman disease is a very rare condition. It may lead to, although extremely rare, serious consequences like blindness and deafness [11–13, 17]. This condition can be treated satisfactorily with surgery and/or corticosteroids [1, 5, 6]. So an early and accurate diagnosis is very important. However, in most of the instances, especially in isolated intracranial RDD, it is commonly mistaken for meningioma, because both of the conditions are usually dura based and their radiological features are almost similar [6, 7]. The only tool that can differentiate the two conditions precisely is histopathology. Unfortunately, the characteristic histological feature (emperipolesis) is not always prominent [3]. So, while diagnosing an intracranial dura based mass, the clinician, radiologist, and pathologist should be cautious about the possibility of Rosai-Dorfman disease.
